# Moxifloxacin and BH3 Mimetic-MIM1 Demonstrate a Potential Synergistic Anti-Melanoma Mode of Action by Cytotoxic and Proapoptotic Activity Enhancement in A375 and G361 Melanoma Cells

**DOI:** 10.3390/molecules30153272

**Published:** 2025-08-05

**Authors:** Artur Beberok, Zuzanna Rzepka, Marta Karkoszka-Stanowska, Dorota Wrześniok

**Affiliations:** Department of Pharmaceutical Chemistry, Faculty of Pharmaceutical Sciences in Sosnowiec, Medical University of Silesia, Jagiellońska 4, 41-200 Sosnowiec, Poland; abeberok@sum.edu.pl (A.B.); zrzepka@sum.edu.pl (Z.R.); marta.karkoszka@sum.edu.pl (M.K.-S.)

**Keywords:** moxifloxacin, BH3-mimetic MIM1, cytotoxicity, apoptosis, melanoma cancer cells

## Abstract

The MIM1-BH3 mimetic, which inhibits the Mcl-1 antiapoptotic protein, may be an efficacious molecule able to induce apoptosis. Previously, we found that moxifloxacin (MXFL) is able to modulate Mcl-1 protein expression. Therefore, in the current study, we assessed the impact of the MXFL, MIM1, and MXFL/MIM1 mixtures on viability and apoptosis in amelanotic A375 and melanotic G361 melanoma cells. The obtained results showed that MXFL and MIM1 exerted high cytotoxic and proapoptotic potential. In the case of two-component models, we have demonstrated that the use of the MIM1 and MXFL mixtures resulted in a significant intensification of both cytotoxic and proapoptotic activity, shown as a modulatory effect on the early and late phases of apoptosis toward the analyzed melanoma cells when compared with MIM1 or MXFL alone. We report, for the first time, the high proapoptotic activity of MIM1 and MXFL applied in a two-component model toward melanoma cells, pointing to the Mcl-1 protein as an important molecular target. The observed potential synergistic mode of action—expressed as cytotoxic and proapoptotic activity enhancement, detected for MIM1 and MXFL—may represent a new direction for further in vitro and in vivo experiments concerning the role of the Mcl-1 protein in the treatment of melanoma. Moreover, the presented results certainly contribute to expanding the knowledge of the pharmacology of both fluoroquinolones and BH3 mimetics, and also enable a better understanding of melanoma cell biology.

## 1. Introduction

Malignant melanoma is the most dangerous skin cancer and is characterized by local invasiveness, recurrence, early metastasis, resistance to therapy, and a high mortality rate [1]. In the USA, the 5-year survival rate for patients with non-metastatic melanoma is high at the level of 98%, while in patients with metastases it decreases to 17% [2]. Cutaneous melanoma represents a significant clinical problem due to its recalcitrance. It encompassed 3–6% of all the new cancer cases in the USA in 2016, excluding basal- as well as squamous-cell carcinomas [3]. A worldwide total of 325,000 new melanoma cases (174,000 males and 151,000 females) and 57,000 deaths (32,000 males and 25,000 females) was estimated for 2020 [4].

Although melanoma accounts for only 4% of all malignant skin cancers, it is responsible for 79% of the deaths caused by cancers of this tissue [5]. One of the reasons for this is the lack of sufficiently effective anti-cancer therapy. Therefore, new solutions are constantly searched for to increase patient survival. In the case of melanoma cells, an increase in the expression of antiapoptotic proteins, including the Mcl-1 protein (Myeloid Cell Leukemia 1), is often observed. This allows the cancer cell to avoid programmed cell death. Therefore, BH3 mimetics (a class of small-molecule drugs designed to mimic the function of BH3-only proteins), (i) being inhibitors of antiapoptotic proteins, can direct the neoplastic cell to the apoptotic pathway; and (ii) may constitute a promising direction in studies concerning new strategies for melanoma treatment [6]. Regarding research in this direction, previously it had been stated that BH3-mimetic MIM1 (4-((*E*)-((*Z*)-2-(cyclohexylimino)-4-methylthiazol-3(2*H*)-ylimino)methyl) benzene 1,2,3-triol), which inhibits the Mcl-1 antiapoptotic protein, may be an efficacious molecule able to induce apoptosis and sensitize melanomas [7,8], as well as glioblastoma multiforme [9] cells, to alkylating agents—dacarbazine (DTIC) and temozolamide.

Antibacterial drugs from the fluoroquinolone group are increasingly being studied in terms of their potential use in cancer chemotherapy [10]. It has been stated that enoxacin (toward A375 and Mel-Ho cells) [11], levofloxacin (toward MCF7, MDA MB-231, MDA MB-468, and SkBr3 cells) [12], ciprofloxacin (toward TK6, WTK1, and NH32 cells) [13], or sparfloxacin (toward C26, HCT116, and HT29 cells) [14] exert high cytotoxic as well as proapoptotic activity. Moreover, it has been demonstrated that fluoroquinolones may enhance the antiproliferative and proapoptotic effect of doxorubicin (for Ewing sarcoma family tumors [15] and prostate cancer cells [16]), etoposide (for THP-1 and Jurkat cells [17]), or docetaxel (for prostate cancer cells [16]). In our latest in vitro study, we have shown that moxifloxacin (MXFL) has potential antiproliferative, cytotoxic, and proapoptotic effects on melanoma cells [18]. Moreover, the analyses carried out so far, including both in in silico and in vitro studies, have shown that fluoroquinolones have the ability to modulate the level of Mcl-1 protein expression, as a common molecular target, with BH3 mimetics. We have demonstrated that MXFL [19] exhibited a capacity to increase the level of the Mcl-1 protein in MDA-MB-231 breast cancer cells. Interestingly, the same direction of modulatory effect was confirmed for MIM1, indicating the Mcl-1 protein as a potential molecular target involved in apoptosis induction [7]. The same phenomenon has also been demonstrated in the case of ciprofloxacin and C32 amelanotic melanoma cells [20]. This suggests that the exposure of cells to MXFL and MIM1 in a two-component model may lead to the induction or strengthening of apoptosis in cancer cells.

Taking into account previously described scientific reports, in the current study, we assessed for the first time the effect of MIM1 and MXFL, alone or in combination, on the viability and apoptosis of melanoma cells. The A375 amelanotic and G361 melanotic melanoma cell lines were used as the experimental model. Moreover, the panel of experiments, including cell viability assessment, was explored by the use of dacarbazine—the gold standard for melanoma treatment.

## 2. Results and Discussion

### 2.1. MXFL and MIM1 Applied in One-Component Model Exert High Cytotoxic Activity

Our recently published results showed that both MXFL [18] and MIM1 [7,8] exhibit significant cytotoxic activity against COLO829 and C32 melanoma cells. In the first part of the current study, the antimelanoma activity of the tested compounds was further evaluated using amelanotic A375 and melanotic G361 melanoma cells as an experimental model, and compared with dacarbazine (DTIC). When analyzing the obtained data for amelanotic A375 melanoma cells (Table 1A–C) and MXFL, it was noticed that the studied drug reduced the number of metabolically active cells by about 47 and 58% only at the highest analyzed concentration (500 µM) after 24 and 48 h of incubation. The lowest values were determined after 72 h treatment, with a decrease in the number of metabolically active A375 cells by about 77%, for MXFL concentration 500 µM. In turn, the exposure of cells to MIM1 caused a significant decrease in the analyzed parameter by about 61, 81, and 82% (treatment with 25, 50, and 100 µM of MIM1 and 24 h incubation time); 63, 87, and 94 (treatment with 25, 50, and 100 µM of MIM1 and 48 h incubation time); and 72, 98 and 98% (treatment with 25, 50, and 100 µM of MIM1 and 72 h incubation time). When comparing the obtained data for DTIC cytotoxic activity, it could be stated that A375 melanoma cells are more sensitive to the MIM1 treatment. A significant reduction in the number of metabolically active cells was observed only at 10, 50, and 100 µM of DTIC and 48 h (decrease by 14, 28, and 35%, respectively) as well as 72 h (decrease by 20, 31, and 35%, respectively) incubation time. Similar observations were made for melanotic G361 melanoma cells (Table 1A–C). However, the observed effect was slightly lower. The highest decline of the viability of the tested cells was observed for MXFL, MIM1, and DTIC in the highest analyzed concentrations (500, 100, and 100 µM, respectively) and 72 h incubation time, with the values of the assessed parameter at the level of 95, 64, and 21%. It is worth emphasizing that the higher cytotoxic activity of MXFL [17] and MIM1 [7,8] toward amelanotic C32 melanoma cells (in contrast to melanotic COLO829 melanoma cells) was previously reported, pointing out that amelanotic melanoma cells are more sensitive to the MXFL and MIM1 treatment. However, it should be noted that the final result could depend on the origin of the cancer cells, the type of cancer, and the response to treatment [21].

### 2.2. Combined Treatment of Melanoma Cells with MXFL and MIM1 Intensified the Cytotoxic Effect

**Figure 1:**
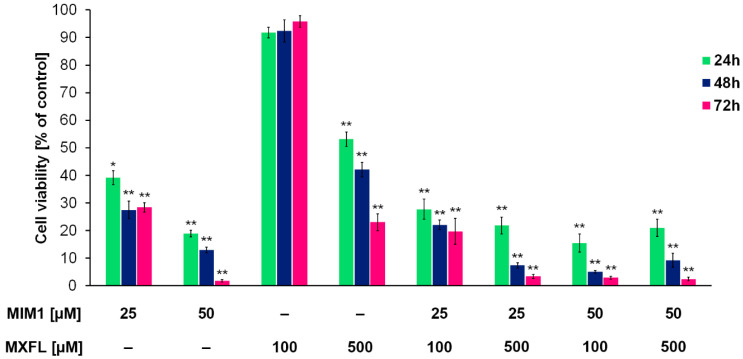
Viability of A375 melanoma cells cultured in the presence of MIM1 and MXFL in one- and two-compound model. The analysis of cell viability—data are expressed as % of the controls; mean values ± SD (*n* = 9). Significance was determined using two-way ANOVA followed by Tukey’s post hoc test. * *p* < 0.5 and ** *p* < 0.05.

**Figure 2:**
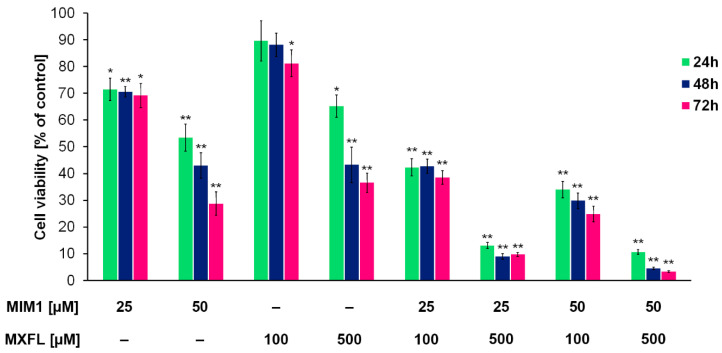
Viability of G361 melanoma cells cultured in the presence of MXFL, MIM1 in one- and two-compound model. The analysis of cell viability—data are expressed as % of the controls; mean values ± SD (*n* = 9). Significance was determined using two-way ANOVA followed by Tukey’s post hoc test. * *p* < 0.5 and ** *p* < 0.05.

Based on the obtained results from the cytotoxic activity of the studied compounds, we decided to use the following concentrations: 25 + 100, 25 + 500, 50 + 100, and 50 µM + 500 µM of MIM1 and MXFL in a two-component experimental model. It was revealed that the combined application of MIM1 and MXFL (Figure 1 and Figure 2) resulted in the decrease in A375 and G361 melanoma cell viability by 72, 78, 84, and 79% (for A375 cells and 24 h incubation time); 78, 92, 95, and 91% (for A375 cells and 48 h incubation time); and 80, 96, 97, and 98% (for A375 cells and 72 h incubation time); and 58, 87, 66, and 89% (for G361 cells and 24 h incubation time); 57, 87, 66, and 89% (for G361 cells and 48 h incubation time); and 61, 90, 75, and 97% (for G361 cells and 72 h incubation time). In the study conducted by Respondek et al. [9], it has been shown that temozolomide demonstrates significantly lower cytotoxic effect, even when applied in the highest concentration (100 μM) and 72 h of incubation time on U87MG glioblastoma cells, when compared to MIM1 alone and MIM1/temozolomide mixture. Moreover, it was revealed that MIM1 and temozolomide used in a two-component model exerted a greater cytotoxic effect on U87MG cells than MIM1 alone, providing, for the first time, convincing evidence that MIM1—which inhibits the antiapoptotic protein Mcl-1—sensitizes glioblastoma cells to the alkylating agent. Similar observations were made in the analysis conducted with the use of MIM1 and DTIC in the C32 and COLO829 experimental model [7,8]. Interestingly, in the current study, we have revealed for the first time that co-treatment of amelanotic A375 and melanotic G361 melanoma cells with MXFL and MIM1 resulted in a greater cytotoxic response of the analyzed cancer cells, indicating a potential synergistic mechanism of action. Moreover, an additional advantage is the reduced risk of adverse events compared to dacarbazine. DTIC is associated with the risk of serious adverse events, including life-threatening events. As with other drugs that inhibit cell division, adverse events may occur in normal tissues with frequent cell division (e.g., bone marrow or epithelial tissue). One of the most common adverse effects of dacarbazine, which limits the possibility of increasing the dose, is the inhibition of hematopoietic function in the bone marrow. There is a risk of leukopenia/neutropenia, sometimes severe, as well as other hematological disorders (e.g., thrombocytopenia or anemia) and infections due to immunocompromised function [22]. In the case of the studied fluoroquinole antibiotics, based on evidence from clinical trials and postmarketing studies, MXFL at standard dosing is generally well tolerated in clinical settings [23]. Common side effects are mild gastrointestinal or neurological complaints, while serious risks like QT prolongation, hepatotoxicity, and severe anemia remain rare but warrant careful monitoring in high-risk populations [23,24,25,26,27].

### 2.3. Combined Treatment of Melanoma Cells with MXFL and MIM1 Augments the Proapoptotic Effect

**Figure 3:**
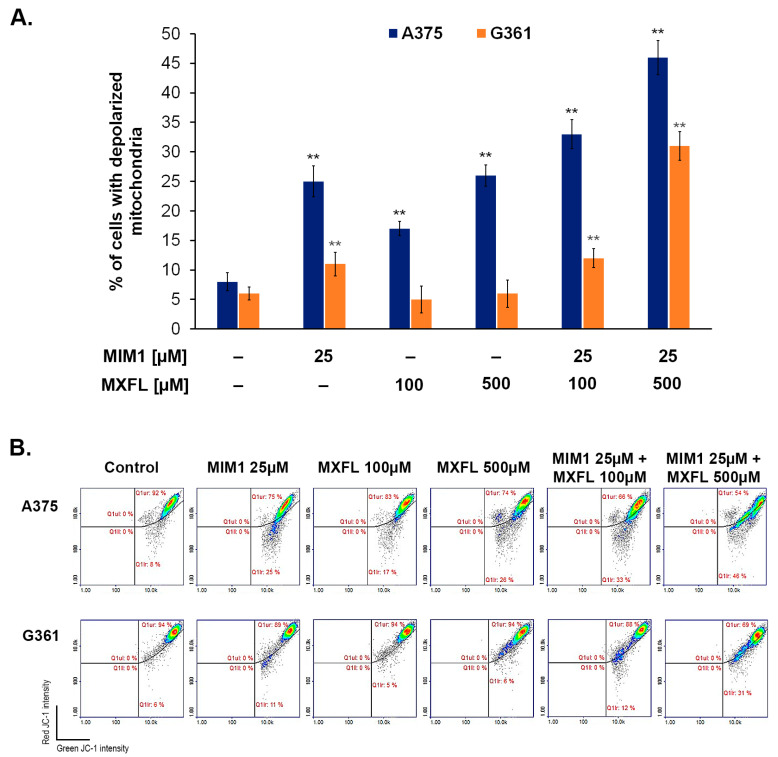
Cytometric analysis of mitochondrial membrane potential assessment. The A375 and G361 melanoma cells were cultured in the presence of MXFL and MIM1 in a one- and two-compound model for 48 h. Significance was determined using two-way ANOVA followed by Tukey’s post hoc test. ** *p* < 0.05 (**A**). Scatter plots presenting changes in the mitochondrial membrane potential in the A375 and G361 melanoma cells treated with MIM1 and MXFL in one- or two-compound model and 48 h incubation time. The graphs are representative of three independent experiments (*n* = 9); Q1ur—cells with polarized mitochondria (healthy); Q1lr—cells with depolarized mitochondria (early apoptotic) (**B**).

**Figure 4:**
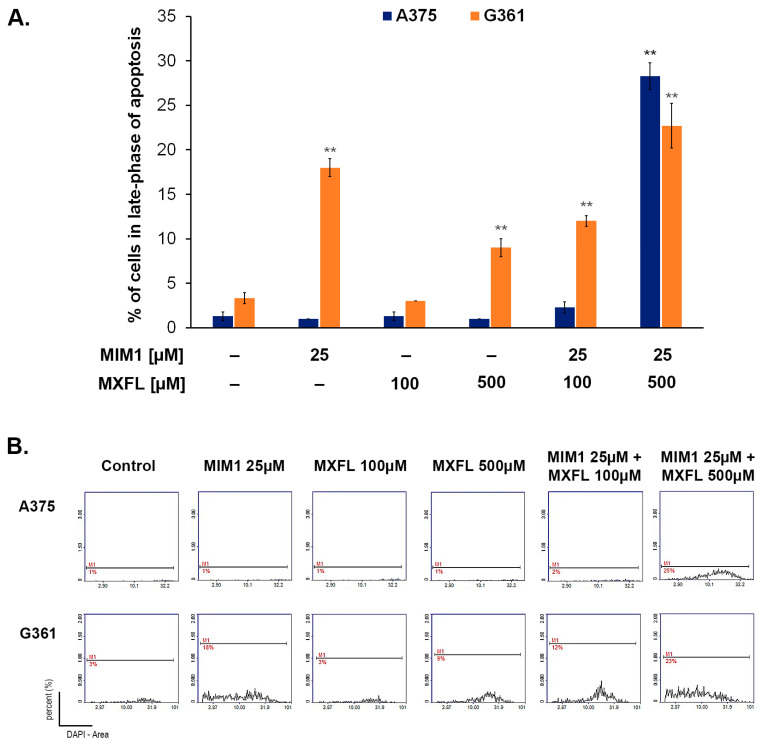
The cytometric analysis of apoptosis late-phase induction in melanoma cells treated with MIM1 and MXFL, in a one- or two-compound model, and 48 h incubation time. Significance was determined using two-way ANOVA followed by Tukey’s post hoc test. ** *p* < 0.05 (**A**). The histograms are representative of three independent experiments; M1—percent of cells in the late phase of apoptosis (**B**).

Encouraged by the data on the cytotoxic activity of MIM1 and MXFL, in the next step of the study, we aimed to assess the proapoptotic activity of the tested compounds when used in single- or dual-compound models. The cytometric analysis of mitochondrial membrane potential (MPP) disruption—one of the key features of mitochondrial-intrinsic apoptotic pathway, revealed that both MIM1 and MXFL caused a significant loss in the measured parameter, with the percentages of depolarized/early apoptotic A375 cells increased from about 8% (control) to 25% (MIM1 25 µM), 17% (MXFL 100 µM), and 26% (MXFL 500 µM) after 48 h incubation time (Figure 3 and Figure 4). In the case of G361 cells, a significant effect was observed only for the tested Mcl-1 inhibitor—MIM1, with an increase in the percentage of depolarized/early apoptotic cells from about 6% (control) to 11%. Moreover, cytometric analysis of apoptosis late-phase induction (DAPI staining, Figure 3 and Figure 4) in melanoma cells showed detectable effects only in the case of melanotic G361 melanoma cells following treatment with MIM1 25 µM (the increase in percentage of late apoptotic cells from 3% to 18%) and MXFL 500 µM (the increase in percentage of late apoptotic cells from 3% to 9%).

BH3 mimetics have been the subject of a large number of clinical trials. Except in a few specific cancer types, which include hematologic malignancies like chronic lymphocytic leukemia and blastic plasmacytoid dendritic cell neoplasm, BH3 mimetics as monotherapy have not produced high response rates [28,29,30]. It should be noted that the most widely studied strategy regarding BH3 mimetics is their combination with current therapies. The main idea behind this approach is that tumors rapidly adapt to current forms of treatment, both conventional chemotherapy and targeted agents, and persisting cancer cells survive, leading to relapse [31]. The primary mechanism by which chemotherapy may act synergistically with BH3 mimetics is by lowering the apoptotic threshold of cells [32]. Preclinical studies have shown synergy between cytotoxic agents such as cytarabine and venetoclax by enhancing BH3 activity and/or suppressing Mcl-1 to induce apoptosis. Thus, venetoclax was associated with deeper remissions in patients with myelogenous leukemia when combined with chemotherapy [33,34]. Based on our earlier in silico and in vitro panel of experiments, we found that MXFL and ciprofloxacin may interact with the Mcl-1 protein as a common molecular target with BH3 mimetic, and this suggests the potential of their synergistic mode of action towards cancer cell with elevated levels of Mcl-1 protein [9,10]. Interestingly, in the present study, we have found that co-treatment of melanoma cells with MIM 1 and MXFL intensified both early and final phases of apoptosis (Figure 3 and Figure 4). This phenomenon was seen after 48 h of incubation for MIM1 25 µM/MXFL 100 µM and MIM1 25 µM/MXFL 500 µM, with an increase in early apoptotic depolarized cells from about 8% to 33% and 46% (A375 cells) as well as 6% to 12 and 31% (G361 cells), respectively. Moreover, for the late-phase apoptosis analysis (Figure 3 and Figure 4), a significant increase in the analyzed parameter was observed for the MIM1 25 µM/MXFL 500 µM mixture (A375 cells)—from 1% to 25%, and MIM1 25 µM/MXFL 100 µM and MIM1 25 µM/MXFL 500 µM mixtures (G361 cells)—from 3% to 12% and 23%, respectively. This observation may indicate that Mcl-1 protein could be considered as a key factor influencing chemosensitivity and programmed cell death.

**Figure 5:**
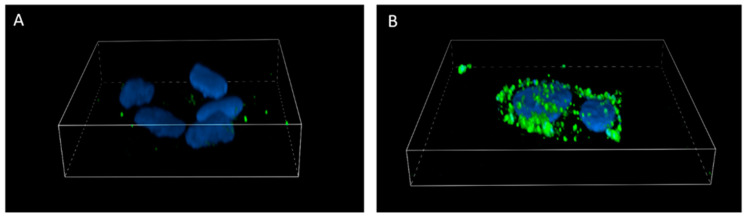
Confocal imaging of A375 melanoma cells (**A**) and cells treated with MIM1 and MXFL mixture (MIM1 25 µM + MXFL 500 µM) for 24 h (**B**).

The molecular aspects of MIM1 and MXFL proapoptotic effect in the two-component model were also analyzed by the visualization of externalized phosphatidylserine (Annexin V assay). The presented confocal imaging (Figure 5) showed that the use of the analyzed compounds in combination (MIM1 25 µM + MXFL 500 µM) after 24 h incubation time caused significant induction of translocation of phosphatidylserine to the outer leaflet of A375 cell membrane—a marker of early apoptotic cells.

It should be noted that the higher proapoptotic activity was observed in amelanotic A375 cells. Melanins, acting as protective molecules with metal chelating properties, may affect the antitumor drug chemosensitivity of melanoma cells [21]. In previous studies conducted by the Cichorek group using Bomirski hamster amelanotic and pigmented transplantable melanomas [35,36,37,38], the differences in biology of these lines were reported. They differed in ultra-structure, metabolism, growth rate, and ability to undergo apoptosis [35,39,40,41,42,43,44]. The authors reported a higher proliferation rate accompanied by decreased ability to undergo spontaneous apoptosis in amelanotic melanoma cells [40,41,45] and suggested that these properties indicate a more aggressive phenotype. However, further studies of this group indicated higher susceptibility of amelanotic melanoma cells to camptothecin-induced apoptosis, with a crucial role of caspases [46]. Similar observations were made in our current study, where the tested compounds used in one- or two-component models exerted higher capacity to induce both the early and late phases of apoptosis in amelanotic melanoma cells when compared with melanotic melanoma cells.

### 2.4. Analysis of the Induction of Morphological Changes in A375 and G361 Melanoma Cells After MIM1 and MXFL Treatment

**Figure 6:**
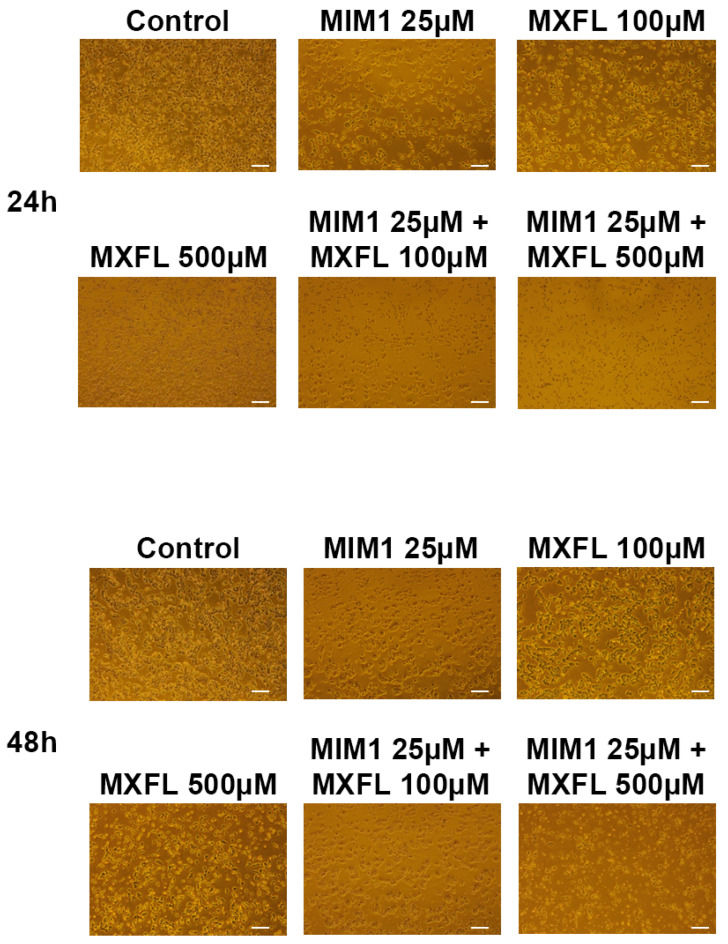
MIM1 and MXFL induce morphological changes in amelanotic A375 melanoma cells. Cells exposed to MIM1 and MXFL in concentrations of 25 µM and 100 µM, and 500 µM, respectively, as well as MIM1 25 µM + MXFL 100 µM and MIM1 25 µM + MXFL 500 µM mixtures for 24 h or 48 h, were observed under a light inverted microscope at 40× magnification (scale bar 100 μm).

**Figure 7:**
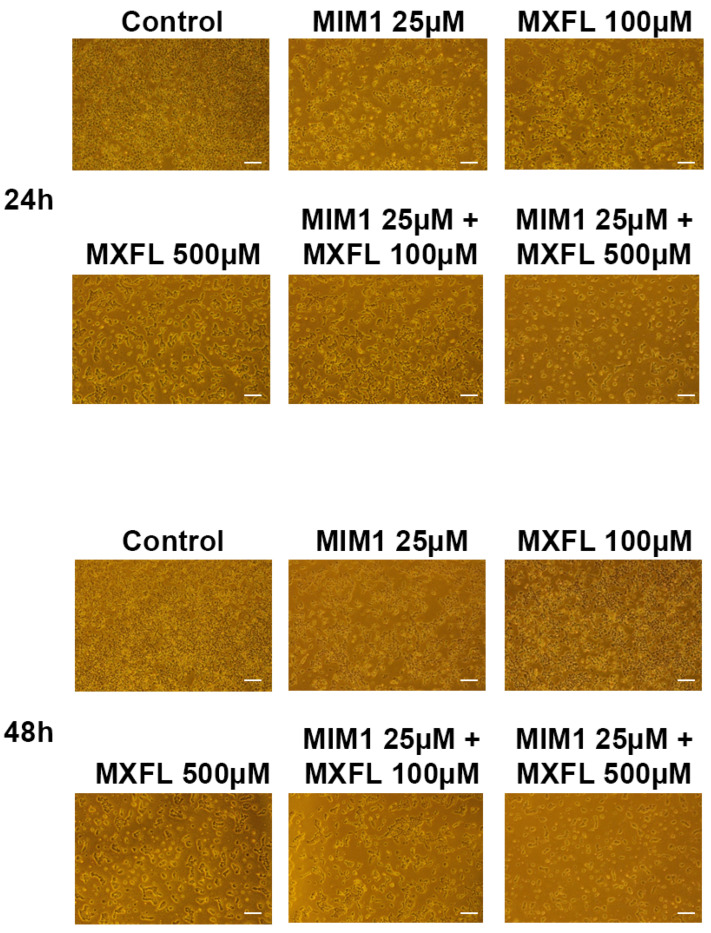
MIM1 and MXFL induce morphological changes in melanotic G361 melanoma cells. Cells exposed to MIM1 and MXFL in concentrations of 25 µM and 100 µM, and 500 µM, respectively, as well as MIM1 25 µM + MXFL 100 µM, MIM1 25 µM + MXFL 500 µM mixtures for 24 h or 48 h were observed under a light inverted microscope at 40× magnification (scale bar 100 μm).

The last assessment of the experimental panel concerned the induction of morphological changes in the A375 and G361 melanoma cells after the MIM1 and MXFL treatment. The morphology of cells was estimated by the use of an inverted light microscope at 40× magnification after exposure of cells to MIM1 and MXFL in concentrations of 25 µM and 100 µM, and 500 µM, respectively, as well as MIM1 25 µM + MXFL 100 µM and MIM1 25 µM + MXFL 500 µM mixtures for 24 h or 48 h. The untreated (control) amelanotic (Figure 6) as well as melanotic (Figure 7) melanoma cells showed all the original properties such as adherent growth, regular shape and size, or cell–cell cohesion. Treatment of cells with the studied compounds induced concentration- and time-dependent irreversible typical apoptotic features, mainly rounding and loss of cell volume. Moreover, loss of cell–cell contact and a decrease in cell number were also observed. These effects intensified significantly when both analyzed cell lines were exposed to MIM1 and MXFL in a two-component model, confirming the enhancement of both cytotoxic and proapoptotic activity.

## 3. Materials and Methods

### 3.1. Cell Culture and Treatment

Human A375 amelanotic and G361 melanotic melanoma cells, purchased from ATCC (CRL-1974TM, ATCC, Manassas, VA, USA), were incubated in a humidified 5% CO2 incubator at 37 °C. The cells were cultured in the recommended medium (DMEM for A375 and McCoys for G361 cells, Sigma Aldrich Inc., St. Louis, MO, USA) supplemented with inactivated fetal bovine serum and the following antibiotics: penicillin (100 U/mL), neomycin (10 μg/mL), and amphotericin B (0.25 μg/mL) (Sigma Aldrich Inc., St. Louis, MO, USA). Treatment with MXFL (Avelox solution for infusion—1 bottle of 250 mL containing 400 mg MXFL as hydrochloride, Bayer Healthcare Pharmaceuticals Inc., Leverkusen, Germany), dacarbazine, or MIM1 (Sigma-Aldrich Inc., Taufkirchen, Germany), alone or in combination with MXFL, was started 24 h after seeding for melanoma cells. The analyzed compound solutions were obtained by diluting the stock solutions in a culture medium. The analysis using the WST-1 assay was made for the compound concentrations ranging from 100 µM to 500 µM for MXFL, from 10 µM to 100 µM for MIM1, and from 10 µM to 100 µM in the case of dacarbazine. Other studies—concerning the assessment of apoptosis—were performed for MXFL and MIM1 at concentrations of 100 μM, 500 µM, and 25 µM in one- or two-component models.

### 3.2. Cell Viability Assessment—WST-1 Assay

The viability of the tested melanoma cells was estimated by the Cell Proliferation Reagent WST-1 (Roche GmbH, Mannheim, Germany). The reagent is a tetrazolium salt (slightly red) that can be reduced in viable cells to a formazan dye by mitochondrial dehydrogenases. In the conducted analysis, 3 × 10^3^ cells per well were placed in a 96-well microplate in a supplemented growth medium and incubated for 24 h. WST-1 was added to cells cultured in 96-well microplates in an amount of 10 µL/well 3 h before the measurement (after 21 h, 45 h, and 69 h). A microplate reader Infinite 200 PRO (TECAN, Männedorf, Switzerland) was used to read absorbance at 440 nm, and 650 nm as a reference wavelength. Control samples were normalized to 100%, and all the tested samples were calculated as a percentage of the control [47].

### 3.3. Mitochondrial Potential Assay

All the experiments were performed using the fluorescence image cytometer NucleoCounter^®^ NC-3000™ (ChemoMetec, Lillerød, Denmark). In the performed analysis, melanoma cells were seeded in T-75 flasks at a density of 2 × 10^6^ cells per flask. The principle of the assay is related to the accumulation of a fluorescent cationic dye JC-1 in the mitochondria in a potential-dependent manner. After the treatment, the cells were suspended in 12.5 µL of Solution 7 (JC-1) and then incubated at 37 °C for 10 min. Next, the cells were centrifuged for 5 min at 400× *g* and washed twice with PBS. The obtained cell pellets were resuspended in 0.25 mL of Solution 8 (DAPI). Finally, the samples were loaded into 8-chamber NC-Slides A8™ and analyzed immediately using the Mitochondrial Potential Assay protocol. The results in the form of scatterplots were used to demarcate the percentage of cells with a polarized or depolarized mitochondrial membrane. The analysis was conducted after 48 h of exposure of cells to the studied compounds.

### 3.4. Annexin-V Assay—Confocal Imaging

Annexin V analysis was conducted using a Nikon Eclipse Ti-E A1R-Si laser confocal microscope with the NIS-Elements AR software (Nikon Instruments, Amsterdam, The Netherlands, version 6.10.01). The cells were seeded in 4-well chamber slides and cultured under standard conditions for 24 h [48]. They were then treated with MXFL and MIM1. After the treatment, the cells were washed with PBS and incubated with a solution containing Hoechst 33342 (ChemoMetec, Lillerød, Denmark) and FITC-labeled annexin V (Biotium, Fremont, CA, USA) in Annexin V Binding Buffer (ChemoMetec, Lillerød, Denmark). This staining allowed visualization of the nuclei and externalized phosphatidylserine, respectively. The samples were incubated for 15 min at 37 °C, then washed twice with Annexin V Binding Buffer. PBS was added to the chambers before observation and imaging. The analysis was conducted after 48 h of exposure of the cells to the studied compounds.

### 3.5. Statistical Analysis

In all the in vitro experiments, mean values were calculated from three separate experiments performed in triplicate (*n* = 9) ± standard error (SD). The results were analyzed using GraphPad Prism 6.01 Software. One-way (early and late phase of apoptosis) and two-way (viability assay) analyses of variance ANOVA were performed, followed by Dunnett’s test and Tukey’s post hoc test, respectively. In all the cases, the statistical significance was set at *p*-value < 0.05.

## 4. Conclusions

Herein, for the first time, we have demonstrated that MXFL and MIM1—the Mcl-1 protein inhibitor used in the two-component model exert high cytotoxic and proapoptotic activity towards A375 and G361 melanoma cells. Moreover, taking into account our previously published data concerning the capacity of MXFL to interact with Mcl-1 protein [10], we have demonstrated that the analyzed fluoroquinolone derivative and MIM1 used in the two-component model exerted a potential synergistic mode of action towards the studied amelanotic and melanotic cancer cells. This phenomenon was shown in both the cytotoxic and apoptosis induction panels of the experiments. However, the current study is the first assessment pointing out that exposure of melanoma cells to MXFL and MIM1 leads to significant enhancement of both the cytotoxic and proapoptotic effects. Therefore, further analysis—including changes in Mcl-1 protein expression—is needed to demonstrate that the studied compounds may act synergistically. Therefore, the current study could (i) serve as the basis for future both in vitro as well as in vivo experiments, (ii) help determine the potential new strategy, and in conclusion, (iii) provide future directions in the treatment of cancers for which Mcl-1 protein is an important molecular target.

## Figures and Tables

**Table 1:** The cytotoxic activity of MIM1, moxifloxacin (MXFL), and dacarbazine (DTIC) on melanoma cells. (**A**) 24 h incubation time. (**B**) 48 h incubation time. (**C**) 72 h incubation time.

**(A)**		
**Studied Compound**	**Cell Viability [% of Control ± SD]**	
	**A375**	**G361**
MIM1 10 µM	97.628 ± 2.343	92.041 ± 4.183
MIM1 25 µM	39.148 ± 2.568	71.422 ± 3.771
MIM1 50 µM	18.839 ± 1.191	53.415 ± 5.110
MIM1 100 µM	17.417 ± 0.873	8.212 ± 1.107
MXFL 50 µM	104.687 ± 2.938	94.407 ± 6.949
MXFL 100 µM	91.781 ± 1.937	89.615 ± 7.542
MXFL 500 µM	53.125 ± 2.581	65.247 ± 4.163
DTIC 10 µM	102.169 ± 5.911	99.872 ± 5.737
DTIC 50 µM	104.864 ± 4.589	96.346 ± 5.502
DTIC 100 µM	98.687 ± 5.378	95.498 ± 6.849
**(B)**		
**Studied compound**	**Cell viability [% of control ± SD]**	
	**A375**	**G361**
MIM1 10 µM	93.111 ± 2.487	96.605 ± 5.145
MIM1 25 µM	27.440 ± 3.221	70.550 ± 1.951
MIM1 50 µM	12.970 ± 0.998	42.984 ± 4.825
MIM1 100 µM	5.598 ± 0.736	10.011 ± 3.877
MXFL 50 µM	102.386 ± 4.728	93.806 ± 6.565
MXFL 100 µM	92.354 ± 3.994	88.092 ± 4.379
MXFL 500 µM	42.118 ± 2.631	43.237 ± 6.606
DTIC 10 µM	85.344 ± 4.856	103.447 ± 6.644
DTIC 50 µM	72.532 ± 2.255	99.229 ± 7.589
DTIC 100 µM	65.160 ± 5.296	87.889 ± 4.129
**(C)**		
**Studied compound**	**Cell viability [% of control ± SD]**	
	**A375**	**G361**
MIM1 10 µM	98.200 ± 3.949	94.190 ± 3.128
MIM1 25 µM	28.381 ± 1.681	69.154 ± 4.555
MIM1 50 µM	1.715 ± 0.431	28.753 ± 4.418
MIM1 100 µM	2.435 ± 0.570	5.262 ± 1.400
MXFL 50 µM	107.138 ± 4.347	88.989 ± 2.524
MXFL 100 µM	95.767 ± 2.090	81.183 ± 4.971
MXFL 500 µM	23.027 ± 3.016	36.551 ± 3.672
DTIC 10 µM	80.430 ± 4.263	95.030 ± 4.417
DTIC 50 µM	68.577 ± 3.806	87.539 ± 3.458
DTIC 100 µM	65.715 ± 2.251	78.907 ± 5.154

## Data Availability

The data that support the findings of this study are available from the corresponding author upon reasonable request.

## References

[1] Slominski R.M., Kim T.-K., Janjetovic Z., Brożyna A.A., Podgorska E., Dixon K.M., Mason R.S., Tuckey R.C., Sharma R., Crossman D.K. (2024). Malignant melanoma: An overview, new perspectives, and vitamin D signaling. Cancers.

[2] Hopkins Z.H., Carlisle R.P., Frost Z.E., Curtis J.A., Ferris L.K., Secrest A.M. (2021). Risk Factors and Predictors of Survival Among Patients with Amelanotic Melanoma Compared to Melanotic Melanoma in the National Cancer Database. J. Clin. Aesthetic Dermatol..

[3] Siegel R.L., Miller K.D., Jemal A. (2016). Cancer statistics, 2016. CA Cancer J. Clin..

[4] Arnold M., Singh D., Laversanne M., Vignat J., Vaccarella S., Meheus F., Cust A.E., de Vries E., Whiteman D.C., Bray F. (2022). Global burden of cutaneous melanoma in 2020 and projections to 2040. JAMA Dermatol..

[5] National Cancer Institute Melanoma of the Skin Cancer Stat Facts. https://seer.cancer.gov/statfacts/html/melan.html.

[6] Belmar J., Fesik S.W. (2015). Small molecule Mcl-1 inhibitors for the treatment of cancer. Pharmacol. Ther..

[7] Respondek M., Beberok A., Rzepka Z., Rok J., Wrześniok D. (2020). Mcl-1 inhibitor induces cells death in BRAF-mutant amelanotic melanoma trough GSH depletion, DNA damage and cell cycle changes. Pathol. Oncol. Res..

[8] Respondek M., Beberok A., Rzepka Z., Rok J., Wrześniok D. (2020). MIM1 induces COLO829 melanoma cell death through mitochondrial membranę breakdown, GSH depletion, and DNA damage. Fundam. Clin. Pharmacol..

[9] Respondek M., Beberok A., Rok J., Rzepka Z., Wrześniok D., Buszman E. (2018). MIM1, the Mcl 1—Specific BH3 mimetic induces apoptosis in human U87MG glioblastoma cells. Toxicol. Vitr..

[10] Yadav V., Talwar P. (2019). Repositioning of fluoroquinolones from antibiotic to anti-cancer agents: An underestimated truth. Biomed. Pharmacother..

[11] Valianatos G., Valcikova B., Growkova K., Verlande A., Mlcochova J., Radova L., Stetkova M., Vyhnakova M., Slaby O., Uldrijan S. (2017). A small molecule drug promoting miRNA processing induces alternative splicing of MdmX transcript and rescues p53 activity in human cancer cells overexpressing MdmX protein. PLoS ONE.

[12] Yu M., Li R., Zhang J. (2016). Repositioning of antibiotic levofloxacin as a mitochondrial biogenesis inhibitor to target breast cancer. Biochem. Biophys. Res. Commun..

[13] Smart D.J., Halicka H.D., Traganos F., Darzynkiewicz Z., Williams G.M. (2008). Ciprofloxacin-induced G2 arrest and apoptosis in TK6 lymphoblastoid cells is not dependent on DNA double-strand break formation. Cancer Biol. Ther..

[14] Gong J.H., Liu X.J., Shang B.Y., Chen S.Z., Zhen Y.S. (2010). HERG K+ channel related chemosensitivity to sparfloxacin in colon cancer cells. Oncol. Rep..

[15] Cornaz-Buros S., Riggi N., DeVito C., Sarre A., Letovanec L., Provero P., Stamenkovic I. (2014). Targeting cancer stem-like cells as an approach to defeating cellular heterogeneity in Ewing sarcoma. Cancer Res..

[16] Pinto A.C., Moreira J.N., Simoes S. (2009). Ciprofloxacin sensitizes hormone-refractory prostate cancer cell lines to doxorubicin and docetaxel treatment on a schedule-dependent manner. Cancer Chemother. Pharmacol..

[17] Fabian I., Reuveni D., Levitov A., Halperin D., Priel E., Shalit I. (2006). Moxifloxacin enhances antiproliferative and apoptotic effects of etoposide but inhibits its proinflammatory effects in THP-1 and Jurkat cells. Biochem. Pharmacol..

[18] Beberok A., Rzepka Z., Respondek M., Rok J., Stradowski M., Wrześniok D. (2019). Moxifloxacin as an inducer of apoptosis in melanoma cells: A study at the cellular and molecular level. Toxicol. Vitr..

[19] Beberok A., Rok J., Rzepka Z., Marciniec K., Boryczka S., Wrześniok D. (2022). Interaction between moxifloxacin and Mcl-1 and MITF proteins: The effect on growth inhibition and apoptosis in MDA-MB-231 human triple-negative breast cancer cells. Pharmacol. Rep..

[20] Beberok A., Rok J., Rzepka Z., Marciniec K., Boryczka S., Wrześniok D. (2020). The role of MITF and Mcl-1 proteins in the antiproliferative and proapoptotic effect of ciprofloxacin in amelanotic melanoma cells: In silico and in vitro study. Toxicol. Vitr..

[21] Slominski R.M., Sarna T., Płonka P.M., Raman C., Brożyna A.A., Slominski A.T. (2022). Melanoma, melanin, and melanogenesis: The Yin and Yang relationship. Front. Oncol..

[22] Medically Reviewed by Drugs.com Last Updated on 25 March 2025. https://www.drugs.com/cons/dacarbazine.html.

[23] Ball P., Stahlmann R., Kubin R., Choudhri S., Owens R. (2004). Safety profile of oral and intravenous moxifloxacin: Cumulative data from clinical trials and postmarketing studies. Clin. Ther..

[24] Haverkamp W., Kruesmann F., Fritsch A., van Veenhuyzen D., Arvis P. (2012). Update on the cardiac safety of moxifloxacin. Curr Drug Saf..

[25] Van Bambeke F., Tulkens P.M. (2009). Safety profile of the respiratory fluoroquinolone moxifloxacin: Comparison with other fluoroquinolones and other antibacterial classes. Drug Saf..

[26] Verma R., Dhamija R., Batts D.H., Ross S.C., Loehrke M.E. (2009). Moxifloxacin induced fatal hepatotoxicity in a 72-year-old man: A case report. Cases J..

[27] Chen W., Peter van Buren P.N. (2017). A case of severe neutropenia from short-term exposure to moxifloxacin. J. Investig. Med. High Impact Case Rep..

[28] Roberts A.W., Davids M.S., Pagel J.M., Kahl B.S., Puvvada S.D., Gerecitano J.F. (2016). Targeting BCL2 with venetoclax in relapsed chronic lymphocytic leukemia. N. Engl. J. Med..

[29] Montero J., Stephansky J., Cai T., Griffin G.K., Cabal-Hierro L., Togami K. (2017). Blastic plasmacytoid dendritic cell neoplasm is dependent on BCL2 and sensitive to venetoclax. Cancer Discov..

[30] Montero J., Haq R. (2022). Adapted to survive: Targeting cancer cells with BH3 mimetics. Cancer Discov..

[31] Montero J., Letai A. (2018). Why do BCL-2 inhibitors work and where should we use them in the clinic?. Cell Death Differ..

[32] Shen S., Vagner S., Robert C. (2020). Persistent cancer cells: The deadly survivors. Cell.

[33] DiNardo C.D., Lachowiez C.A., Takahashi K., Loghavi S., Xiao L., Kadia T. (2021). Venetoclax combined with FLAG-IDA induction and consolidation in newly diagnosed and relapsed or refractory acute myeloid leukemia. J. Clin. Oncol..

[34] Chua C.C., Roberts A.W., Reynolds J., Fong C.Y., Ting S.B., Salmon J.M. (2020). Chemotherapy and venetoclax in elderly acute myeloid leukemia trial (CAVEAT): A phase Ib dose-escalation study of venetoclax combined with modified intensive chemotherapy. J. Clin. Oncol..

[35] Bomirski A., Slominski A., Bigda J. (1988). The natural history of a family of transplantable melanomas in hamsters. Cancer Metastasis Rev..

[36] Bomirski A., Wrzolkowa T., Arendarczyk M., Bomirska M., Kuklinska E., Slominski A. (1987). Pathology and ultrastructural characteristics of a hypomelanotic variant of transplantable hamster melanoma with elevated tyrosinase activity. J. Investig. Dermatol..

[37] Bomirski A., Dominiczak T., Nowinska L. (1962). Spontaneous Transplantable Melanoma in the Golden Hamster (Mesocricetus Auratus). Acta Unio Int. Contra Cancrum..

[38] Bomirski A., Zawrocka-Wrzolkowa T., Pautsch F. (1971). Ultrastructure of transplantable melanotic and amelanotic melanoma of Hamsters. Acta Med. Pol..

[39] Slominski A., Paus R. (1993). Bomirski melanomas—A versatile and powerful model for pigment cell and melanoma research. Int. J. Oncol..

[40] Cichorek M., Kozlowska K., Wachulska M., Zielinska K. (2006). Spontaneous apoptosis of melanotic and amelanotic melanoma cells in different phases of cell cycle: Relation to tumor growth. Folia Histochem. Cytobiol..

[41] Kozlowska K., Zarzeczna M., Cichorek M. (2001). Sensitivity of transplantable melanoma cells to cytokines with regard to their spontaneous apoptosis. Pathobiology.

[42] Kozlowska K., Cichorek M., Zarzeczna M., Brozek J., Witkowski J.M. (2002). Heterogeneous susceptibility to spontaneous and induced apoptosis characterizes two related transplantable melanomas with different biological properties. Pigment. Cell Res..

[43] Scislowski P.W., Slominski A., Bomirski A. (1984). Biochemical characterization of three hamster melanoma variants–II. Glycolysis and oxygen consumption. Int. J. Biochem..

[44] Slominski A., Scislowski P.W., Bomirski A. (1984). Biochemical characterization of three hamster melanoma variants–I. Tyrosinase activity and melanin content. Int. J. Biochem..

[45] Cichorek M., Zarzeczna M., Kozlowska K. (2002). Tumoricidal effect of macrophages on transplantable melanoma cells with regard to their sensitivity to exogenous cytokines. Folia Histochem. Cytobiol..

[46] Cichorek M., Kozlowska K., Bryl E. (2007). The activity of caspases in spontaneous and camptothecin-induced death of melanotic and amelanotic melanoma cell. Cancer Biol. Ther..

[47] Marciniec K., Rzepka Z., Chrobak E., Boryczka K., Latocha M., Wrześniok D., Beberok A. (2023). Design, synthesis and biological evaluation of quinoline-8-sulfonamides as inhibitors of the tumor cell-specific M2 isoform of pyruvate kinase: Preliminary study. Molecules.

[48] Karkoszka M., Rok J., Kowalska J., Rzepka Z., Banach K., Wrześniok D. (2024). Phototoxic action of meloxicam contributes to dysregulation of redox homeostasis in normal human skin cells—Molecular and biochemical analysis of antioxidant enzymes in melanocytes and fibroblasts. Toxicol. Vitr..

